# Comparative transcriptomic and molecular biology analyses to explore potential immune responses to *Vibrio parahaemolyticus* challenge in *Eriocheir sinensis*

**DOI:** 10.3389/fcimb.2024.1456130

**Published:** 2024-12-20

**Authors:** Duanduan Chen, Yunteng Xin, Jian Teng, Xiaodong Zhao, Jianbiao Lu, Yubao Li, Hui Wang

**Affiliations:** ^1^ Phage Research Center of Liaocheng University, Liaocheng, China; ^2^ Shandong Agricultural University, Taian, China; ^3^ Yantai Jinghai Marine Fishing Co, Yantai, China; ^4^ Shandong Vocational Animal Science and Veterinary College, Weifang, China

**Keywords:** Eriocheir sinensis, vibrio parahaemolyticus, RNA-seq, immunomodulatory mechanisms, immune response

## Abstract

*Vibrio parahaemolyticus* is a significant pathogen affecting shrimp and crab farming, particularly strains carrying genes associated with acute hepatopancreatic necrosis syndrome. However, the immune response of *Eriocheir sinensis* to *V. parahaemolyticus* infection remains unclear. To address this knowledge gap, an experiment was conducted to establish a *V. parahaemolyticus* infection model. This model aimed to compare pathological damage and enzyme activity changes in *E. sinensis* hepatopancreas tissue at various infection time points, and to examine transcriptome changes in individuals exhibiting different clinical symptoms of infection. The results showed that intramuscular injection of 1.78 × 10^6^ CFU/mL of *V. parahaemolyticus* for 24 hours resulted in a 50% mortality rate among the experimental animals. Pathological findings revealed that the infection led to a change in color of the hepatopancreas tissue from bright yellow to white, diffuse tissue cell distribution, and hepatopancreatic necrosis. Additionally, there was a significant increase in the activities of alanine aminotransferase and aspartate aminotransferase in the hepatopancreas (*P* < 0.01). Furthermore, the activities of superoxide dismutase, total antioxidant capacity, phenoloxidase, alkaline phosphatase, and acid phosphatase initially increased and then decreased. RNA-seq analysis revealed 11,662 differentially expressed genes compared to the susceptible group and control group, with 6,266 genes up-regulated and 5,396 genes down-regulated. When comparing the susceptible group to the disease-resistant group, 13,515 differentially expressed genes were identified, with 7,694 genes up-regulated and 5,821 genes down-regulated. Finally, comparison between the disease-resistant group and control group yielded 13,515 differentially expressed genes, with 7,631 genes up-regulated and 3,111 genes down-regulated. Differential gene enrichment analysis revealed pathways such as phagosomes, cancer pathways, proteoglycans in cancer, ribosomes, protein processing in the endoplasmic reticulum, starch and sucrose metabolism, and lysosome signaling pathways. Furthermore, 342 immune-related genes with differential expression were identified, primarily enriched in 22 pathways linked to cell signaling. These genes play a crucial role in defense against bacterial invasion and immune response regulation through various signaling pathways. Overall, this study provides valuable insights into the defense mechanisms and understanding of Chinese mitten crab immunity against bacterial infection by examining changes in mRNA, enzyme activity, and hepatopancreatic damage during infection.

## 1 Introduction

Chinese mitten crabs (*Eriocheir sinensis*) are a valuable aquaculture species in East Asia (Sun et al., 2013; Qin et al., 2024). However, the expansion of intensive aquaculture models has created new opportunities for the crab farming industry but has also led to frequent occurrences of bacterial (Wang, 2011; Tian et al., 2024), viral (Zhang et al., 2004; Lorgen-Ritchie et al., 2023), and parasitic diseases (Li et al., 2011; Xin et al., 2024). These diseases have resulted in significant decreases in farm production and substantial economic losses (Sun et al., 2013) Although bacterial diseases in crabs are less common than viral and protozoan diseases (Wang, 2011), research has primarily focused on marine crabs that develop bacteremia or bacterial diseases affecting the exoskeleton (Krantz et al., 1969; Sizemore et al., 1975; Davis and Sizemore, 1982; Coates and Rowley, 2022). Previous studies on *Callinectes sapidus*, *Callinectes bocourti* (Wang, 2011), and *Cancer irroratus* (Newman and Feng, 1982) have observed significant reductions in blood cell counts and intravascular coagulation following *Vibrio* infection, with endotoxins in the bacterial cell wall penetrating tissues (Sritunyalucksana and Söderhäll, 2000; Jin et al., 2020; Zhang et al., 2022). Several studies have demonstrated the ability of *Vibrio parahaemolyticus (V. parahaemolyticus)* to infect Chinese mitten crabs and their offspring (Liu et al., 2020). Further research on the infection process and the harm caused by *V. parahaemolyticus* to Chinese mitten crabs is crucial for enhancing the overall health of crab breeding practices.

The extensive range of genes and virulence factors of *Vibrio* splays a crucial role in its heightened pathogenicity, which allows it to successfully invade a variety of host species, causing severe damage while evading host defenses. One highly dangerous species is *V. parahaemolyticus*, which poses a threat to both humans and aquatic animals in freshwater, estuarine, and marine environments (Baker-Austin et al., 2018). The genomes of *V. parahaemolyticus* exhibit a high level of diversity, with two chromosomes, frequent horizontal gene transfer, and high recombination rates (Martinez-Urtaza and Baker-Austin, 2020). Toxigenic strains of *V. parahaemolyticus* produce various virulence factors, such as the heat-stable direct haemolysin (TDH) and the TDH-associated haemolysin, which are responsible for Kanagawa hemolysis. The encoding genes for these toxin factors are *tdh* and *trh*, respectively (Okuda et al., 1997). Nonetheless, strains lacking hemolysins can also be associated with human infections (Martinez-Urtaza and Baker-Austin, 2020). In the aquaculture process, we concentrate on specific strains that have the potential to cause diseases in aquatic animals.

*V. parahaemolyticus* has been expanding globally, with epidemics often linked to coastal warming (Baker-Austin et al., 2018). In 2021, a specific strain of *V. parahaemolyticus* emerged in Asia, primarily impacting crustaceans, particularly shrimp (Powers et al., 2021). Infection with this bacterium, referred to as acute hepatopancreatic necrosis disease (AHPND) or early mortality syndrome (EMS), has been extensively researched and found to carry the binary toxins PirA *Vp* and PirB *Vp* in the extrachromosomal virulence plasmid pVA1 (Tran et al., 2013; Lee et al., 2015; Thitamadee et al., 2016; Vandeputte et al., 2024). Clinical indications of AHPND in shrimp include a damaged hepatopancreas, soft shells, discontinuous or atrophic gut, absence of contents, and pale discoloration, often resulting in mortality rates as high as 100% (Han et al, 2015). Our previous research has shown that this strain has the potential to infect freshwater crustaceans (Wu et al., 2017; Chen et al., 2021). Given that the Chinese mitten crab is a migratory and reproducing crustacean, it is also vulnerable to infection. Hence, it is imperative to conduct additional research on the infection mechanism and impact of *V. parahaemolyticus* on Chinese mitten crabs.

The protection of multicellular organisms from invasive species has greatly relied on the evolution of the immune system. To resist the invasion of pathogenic microorganisms, two effective immune systems have evolved: innate immunity and adaptive immunity (Lee and Söderhäll, 2002). Invertebrates exhibit limited adaptive immunity and primarily depend on innate immunity to combat pathogen invasion (Loker et al., 2004; Schulenburg et al., 2007; Tong et al., 2022). *E. sinensis*, being an invertebrate, predominantly utilizes innate immune mechanisms, such as agglutination, encapsulation, phagocytosis, coagulation proteins, and bactericidally active peptides (Vazquez et al., 2009). The humoral immune system activates the phenol oxidase (ProPO) system, which leads to the production of antimicrobial peptides (AMPs). Cellular immune responses primarily involve phagocytosis and encapsulation of pathogenic bacteria (Lemaitre et al., 1995; Lemaitre and Hoffmann, 2007; Chen et al., 2025). The current understanding of the immune regulation of Chinese mitten crabs in response to *V. parahaemolyticus* infection is limited. Further investigation is needed to explore the immune response of Chinese mitten crabs at various stages of *Vibrio* infection and analyze the differential transcription of the transcriptome.

*E. sinensis*, a migratory crustacean, is susceptible to infection by *Vibrio*, particularly *V. parahaemolyticus*, which carries the *priA*/*priB* toxin gene. This study aims to develop an animal infection model using molecular biology and RNA-seq technologies. The model will be used to comprehensively analyze the changes in body fluid enzyme activity factors, hepatopancreas damage, and hepatopancreas function of *E. sinensis* during the infection process with *V. parahaemolyticus*. Furthermore, the study will investigate the differential expression of transcriptome genes, immune response, and changes in body indicators of experimental animals during the infection process. The findings from this study will contribute to a better understanding of the infection process of *V. parahaemolyticus* and the host’s immune mechanism.

## 2 Materials and methods

### 2.1 Information about test animals and V. parahaemolyticus

Specimens of *E. sinensis* were acquired from Baodao Agricultural Technology Co., Ltd., located in Dongping County, Shandong Province, which specializes in aquaculture. These crabs had a mean weight of 15 ± 0.5 grams. They were housed in a laboratory tank equipped with a circulating water system and aerated with tap water. The temperature of the water was maintained at 26 ± 1.5°C. To provide hiding spots, PE pipes and artificial water plants were placed within the tank. Prior to the experiment, the crabs were acclimated in the laboratory for a duration of 1 week. Pre-experiments and acute infection experiments were conducted once the crabs’ state was stable. Prior to the commencement of the experiment, we randomly selected 10 experimental animals for the assessment of *V. parahaemolyticus*. The results indicated that these experimental animals did not harbor *V. parahaemolyticus*. Throughout the acclimation period, the crabs were provided with commercial crab feed; however, feeding was ceased on the day preceding the experiment. Specifically, the strain of *V. parahaemolyticus* was isolated from red claw crayfish culture ponds.

The *V. parahaemolyticus* strain utilized in this experiment was obtained, purified, and identified by the Research Laboratory of Aquatic Animal Health Breeding within the College of Animal Science and Technology at Shandong Agricultural University. This strain is stored at the Phage Research Centre within the College of Agriculture at Liaocheng University. The whole genome analysis of the strains utilized has been completed, and the original sequencing data are stored in the National Center for Biotechnology Information (NCBI) under the storage number PRJNA902323. To culture the strains, overnight growth was achieved on citrate bile salt sucrose agar sulphate plates (TCBS, Cat: HB4130, Qingdao Hope Biotechnology Co., Ltd., China). Single colonies were then transferred to LB broth (LB, Cat: HB0128, Qingdao Hope Bio-Technology Co., Ltd., China) and incubated for 6 hours. Subsequently, 1 mL of broth was centrifuged at 4000 rpm for 5 minutes, the supernatant was discarded, and 50 μL of RNase-free water was added. To detect the virulence genes carried by the experimental strains, PCR was used. After amplification and sequencing of the PCR products, we performed BLAST comparative analysis on all valid PCR results using the NCBI database. The sequences of all virulence genes can be found in **Table 1**. The primer sequences pertinent to this article are also listed in **Table 1**.

**Table 1:** Primer sequences.

Gene ID	FORWARD (5’-3’)	REVERSE (5’-3’)
*GyrB*	GAAGGBGGTATTCAAGC	GAGTCACCCTCCACWATGTA
*TnaA*	TGTACGAAATTGCCACCAAA	AATATTTTCGCCGCATCAAC
*PyrC*	AGCAACCGGTAAAATTGTCG	CAGTGTAAGAACCGGCACAA
*RecA*	GAAACCATTTCAACGGGTTC	CCATTGTAGCTGTACCAAGCACCC
*LuxR*	ATCTTGCGGCGTGTAGTG	ATCTTGCGGCGTGTAGTG
*pirA*	TGACTATTCTCACGATTGGACTG	CACGACTAGCGCCATTGTTA
*pirB*	TGATGAAGTGATGGGTGCTC	TGTAAGCGCCGTTTAACTCA
*CDSP*	CTGGATGGCTCTGCTTGGCTAC	CGACCACTTGTCCGAGGCTATTG
*Integrin*	AGTGGATGTGGCTTGCTTGTAGATG	GGCGTTGCTACTCATGTGGAAGG
*serine/threonine protein kinase(SP)*	GCATCCAAAGGGAAGCCAGAAATTG	TGAGGAACCTGTGGGAGAGAAGC
*LGBP*	CGACTTCTGGAAAGGGCGTGAC	CCACACCTTCACATAGTCCACCTTC
*ALF*	GAACGACCACTCCCTCTCAACAATC	ACCGAACAGAACGCTCCTCCTC
*HSP90*	CACTCGCAGTTCATTGGCTATCCC	TCTTGGGCTTCTCATCCTCTTCCTC
*Relish*	AAGATTGACGAGGAGGCTGAGGAG	GCAGGGTGATGGGTCGGTAATTC
*GTP*	AGAAGATGAAGCAAGAGCCTGTGAG	CAAACACCTCCCGAACACCTTCC
*AKP*	GCAACATTCCCGAGGAGTGCAA	ACCCCAGTCTTCGTCTATGG
*Toll*	GCACATTCTTCCCTCACCCTATCAC	TGCTTCTCTTGCTTCTGGCTTACTG
*HC*	TGCCTGGTTCTGTTCGCTCTGG	AGGGCTGGGTCACGGATGTCTT
*LZM*	TACTGGTACTGGTACGACGGCGGAGAG	CATCATCGGCGGTCACGTATCGGTTCA
*SOD*	CGAGCTGTCTGGCATTGAGGTG	CCCTGCCTGCTTCACTTGGATG
*β-actin*	GGCATCCACGAGACCACTTACAAC	GCGAGGGCAGTGATTTCCTTCTG

### 2.2 Modeling of infection and sample collection

A total of 100 specimens of Chinese mitten crabs were randomly distributed into 5 groups, each comprising 20 individuals. Cultivated strains (100 mL) were incubated overnight at a temperature of 37°C, after which they were centrifuged at a speed of 4000 rpm for 10 minutes using a high-speed centrifuge. The supernatant was discarded, and the strains were washed three times using a phosphate buffer solution. The concentration of the bacterial solution was measured using an ultra-microspectrophotometer and subsequently diluted with a buffered salt solution to obtain concentrations of 1×10^5^, 1×10^6^, 1×10^7^, and 1×10^8^ CFU/mL. Each experimental subject was administered an injection of 50 μL of the bacterial solution, whereas the control group received an injection of the same volume of phosphate-buffered saline (PBS).

The mortality rate of the experimental group was recorded at various intervals following the initiation of the disease (**Supplementary Table S1**), and the LD50 concentration of the pathogenic strain was determined to be 1.78×10^6^ CFU/mL after 24 hours using this methodology (Saganuwan, 2016). In the subsequent stage, 200 experimental subjects were arbitrarily selected and divided equally into two groups: the experimental group and the control group, each containing 100 specimens. The experimental group was administered an injection of 50 μL of fresh bacterial solution at the LD50 concentration, whereas the control group was administered an injection of the same volume of phosphate-buffered saline (PBS).

Samples of tissue from the hepatopancreas were procured at intervals of 6, 12, 24, 48, and 72 hours after the infection. Based on clinical symptoms and behavioral observations of Chinese mitten crabs, individuals exhibiting significant pathological conditions within 6 hours after challenge were categorized as the susceptible group (Es_SC), whereas those in good health or displaying no apparent symptoms after 24 hours were classified as the disease-resistant group (Es_AI). The individuals injected with PBS were designated as the control group (Es_CG). When collecting samples, begin by using 75% alcohol-soaked cotton balls to thoroughly wipe the entire body of the experimental animals. Once all samples have been obtained, they should be rapidly frozen in liquid nitrogen to preserve their freshness, and stored in a refrigerator at -80°C for subsequent assays to determine immunoenzymatic activity and extract RNA, respectively. Additionally, hepatopancreatic tissues were collected at different intervals after rinsing with phosphate-buffered saline and fixing them with a solution of 4% paraformaldehyde. Pathological sections were prepared to observe any changes in pathology (Zhang et al., 2020).

### 2.3 Histopathological analysis

Histopathological analysis was conducted on the hepatopancreas of *E. sinensis* at 6, 24, and 72 hours after infection, with the PBS injection group serving as the control. The hepatopancreas was fixed with paraformaldehyde for 24 hours and then embedded in paraffin blocks. Subsequently, the tissue was dehydrated using different concentrations of ethanol. Using a microtome, the blocks were sectioned into thicknesses of 5-6 μm. The resulting sections were stained with hematoxylin and eosin and subsequently examined under light microscopy following fixation with resin.

### 2.4 Detection of humoral immunoenzymatic activity

To detect humoral immunoenzymatic activity, tissue samples were thawed from the -80°C refrigerator. Filter paper was de-watered, weighed, and cut appropriately with scissors. The cut tissue samples were placed in sterile 1.5 mL centrifuge tubes and mechanically ground on ice using a grinder. After spinning at 2500 revolutions per minute (rpm) for 10 minutes, the liquid above was discarded. Subsequently, the tissue was mixed with saline solution to produce a 1% solution of crushed tissue for examining the activities of immune-related enzymes. The primary immune-related enzymes evaluated included acid phosphatase (ACP: A060-2-2), peroxidase (PO: A084-1-1), total antioxidant capacity (T-AOC: A015-2-1) alkaline phosphatase (AKP/ALP: A059-3-1), alanine transaminase (GPT/ALT: C009-1-1), aspartate transaminase (AST/GOT: C010-2-1), Lysozyme (LZM: A050-1-1) and superoxide dismutase (SOD: A001-4-1). These kits were procured from Nanjing Jiancheng Bioengineering Institute in China (http://www.njjcbio.com/) and were used according to the manufacturer’s instructions. Three biological replicates were conducted for each sample.

### 2.5 RNA sequencing grouping

RNA was extracted from three samples randomly selected from a total of nine collected samples, which were maintained at low temperatures throughout the process. Total RNA extraction from crabs was performed according to the manufacturer’s instructions using TRIzol (Invitrogen, USA). Each high-quality, non-degraded RNA sample underwent library construction using the NEBNext^®^ Ultra™ RNA Library Preparation Kit (NEB, USA) with 1 μg per sample. The HiSeq 4000 platform (Illumina, USA) was used to generate 150 bp paired-end (PE) reads. RNA-Seq analysis and clustering were outsourced to Novogene Biotechnology Co., Ltd. in Beijing, China, which employs Illumina sequencing technology. Initial processing of raw data (raw reads) in fastq format was performed using an in-house perl script.

### 2.6 Data quality control and bioinformatics analysis

For RNA-seq data without a reference genome, data quality control was performed with the Trimmomatic software (Bolger et al., 2014) to remove adapters and low-quality reads (N content >5%, base mass values <20). Clean reads were obtained and then assembled using Trinity (Grabherr et al., 2011) to generate a reference sequence for subsequent analysis.

To identify genes that are under-expressed or only partially detected across samples, all samples from the same species were combined to achieve a comprehensive assembly result for subsequent analyses, such as differential expression analysis. However, samples from different species underwent separate assemblies due to significant genomic differences. In the absence of a reference genome, transcriptome analysis involved assembling the obtained sequences into transcripts and utilizing the Corset software (Davidson and Oshlack, 2014) for hierarchical clustering. The clustered sequences served as a reference for subsequent analyses, including quality control, annotation using seven databases (NR: NCBI non-redundant protein sequences; NT: NCBI nucleotide sequences; Kyoto Encyclopedia of Genes and Genomes) KEGG: http://www.genome.jp/kegg/; SwissProt: http://www.ebi.ac.uk/uniprot/; PFAM: http://pfam.sanger.ac.uk/; GO: http://www.geneontology.org; KOG: http://www.ncbi.nlm.nih.gov/COG/), quantification, differential expression analysis, and functional enrichment.

### 2.7 RT-qPCR validation

The 2^-ΔΔCt^ was employed to compute the relative gene expression (Livak and Schmittgen, 2001). In this investigation, we assessed the expression levels of four genes in diverse samples via RT-qPCR, examining gene alterations during infection. To facilitate the comparison of detected samples during the exponential phase of the RT-qPCR reaction, it is essential to first establish a specific fluorescence signal threshold. Typically, this threshold is determined based on the fluorescence signals from the initial 15 cycles of the PCR reaction, which serve as the fluorescence background signal. If a detected fluorescence signal surpasses this threshold, it is regarded as a true signal and can be utilized to define the threshold cycles (Ct) for the sample. There exists a linear inverse relationship between the Ct value of each template and the logarithm of the starting template’s copy number in the sample; thus, a higher starting copy number corresponds to a lower Ct value. Each specimen was analyzed in triplicate, and the Ct values were averaged. The primer sequences employed in this study are listed in **Table 1**.

### 2.8 Statistical analysis

Most data analyses were conducted using software or built-in algorithms within the software packages. Feature enrichment tests were carried out using the GOseq package, while differential expression analyses were performed using the DESeq2 package. The criteria used for screening differentially expressed genes were | log_2_(Fold Change) | > 1, with a padj value of less than 0.05 (*P* < 0.05). considered statistically significant.

To assess the significance of differences between the means of two datasets, Independent Student’s t-tests were performed using SPSS version 22.0. This analysis included comparisons between RT-qPCR and RNA-seq data, with a p-value below 0.05 (*P* < 0.05). indicating statistical significance, a p-value below 0.01 (*P* < 0.01). indicating high significance, and a p-value above 0.05 (*P* > 0.05) indicating no significance.

## 3 Result

### 3.1 Information on *V. parahaemolyticus* strain

**Figure 1:**
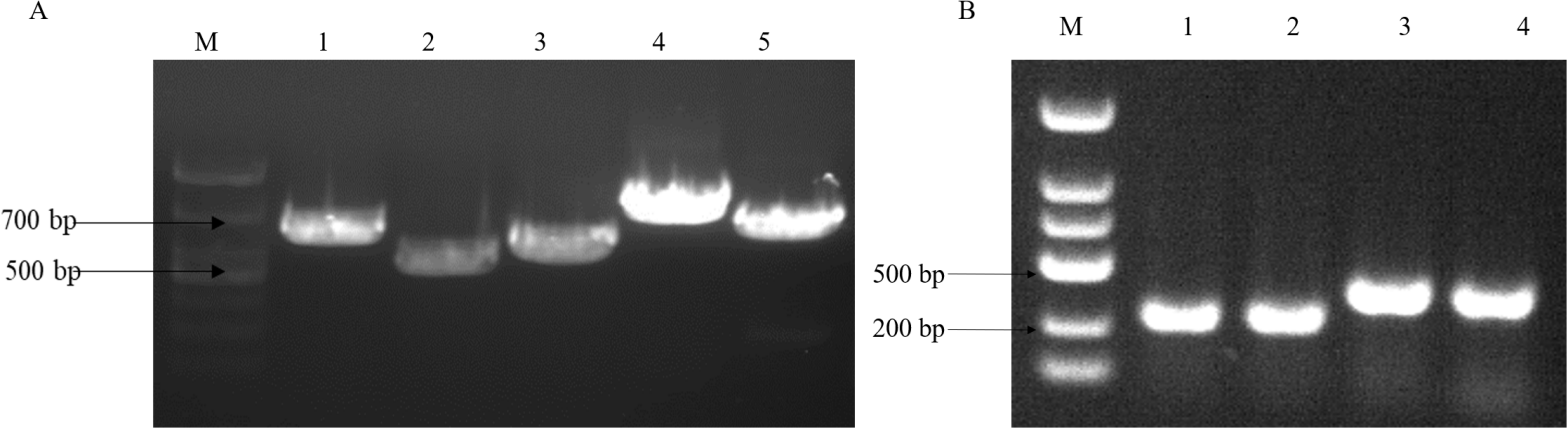
V*. parahaemolyticus* virulence gene gel electrophoresis. **(A)** M:2000bp marker, lane1: *Gyr gene* (629bp), lane2: *TnaA gene* (463bp), lane3: *PyrC gene* (533bp), lane4: *RecA gene* (773bp) and lane5: *LuxR gene* (679bp); **(B)** lane1.2: *PirA gene* (284bp), lane 3.4: *PirB gene* (392bp).

To analyze the virulence gene present in the strains, we conducted a gel electrophoresis (PCR) analysis. The cultured fresh colonies were identified using PCR, confirming the presence of several virulence genes: *Gyr* (629bp), *TnaA* (463bp), *PyrC* (533bp), *RecA* (773bp), and *LuxR* (679bp). Additionally, the virulence genes *PirA* (284bp) and *PirB* (392bp), which are associated with pancreatic necrosis, were also detected (**Figure 1**) (Cheng and Wang, 2022). The PCR products were sequenced by a sequencing company and subsequently compared with known virulence gene sequences of *V. parahaemolyticus* in the NCBI database. The results indicated that all identified virulence genes were pathogenic genes associated with *V. parahaemolyticus.* The antibacterial susceptibility test revealed that the macrolide antibiotic erythromycin exhibited no activity against the bacteria (**Table 2**).

**Table 2:** Drug sensitivity test results for *V. parahaemolyticus*.

Type of medicine	Name of medicine	Diameter of inhibition circle(mm)
Aminoglycoside antibiotics	Gentamicin	18.3 ± 0.3
	Amikacin	18.5 ± 0.3
Quinolone antibiotics	norfloxacin	19.8 ± 0.3
	Ciprofloxacin	28.9 ± 0.3
Macrolide antibiotics	Erythromycin	0
Tetracycline antibiotics	Tetracycline	29.2 ± 0.3
Cephalosporin antibiotics	Cephalosporins	17.1 ± 0.3
	Cefuroxime	14.9 ± 0.3
Penicillin antibiotics	Ampicillin	11.9 ± 0.3
	Piperacillin	21.1 ± 0.3
Sulfonamide antibiotics	Cotrimoxazole	18.1 ± 0.3
	Chloramphenicol	23.2 ± 0.3

### 3.2 Determination of LD50 and histopathological changes of hepatopancreas

**Figure 2:**
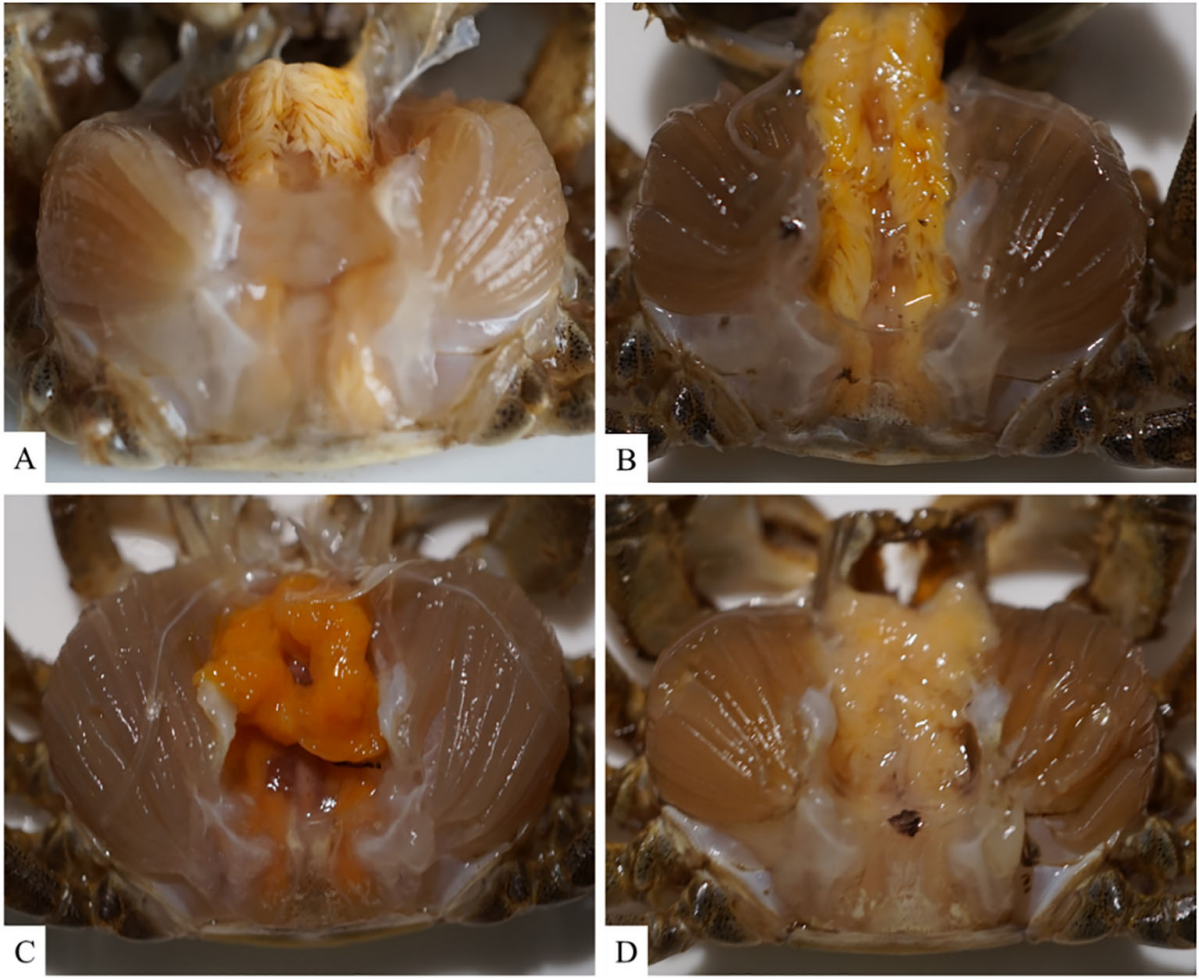
Illustrates the clinical histological changes in the hepatopancreas of *Eriocheir sinensis* resulting from *Vibrio parahaemolyticus* infection at various time points. **(A)** depicts the anatomy of a healthy crab from the control group, while **(B–D)** present the anatomy of the hepatopancreas in diseased crabs at 6, 24, and 72 hours post-infection, respectively.

**Figure 3:**
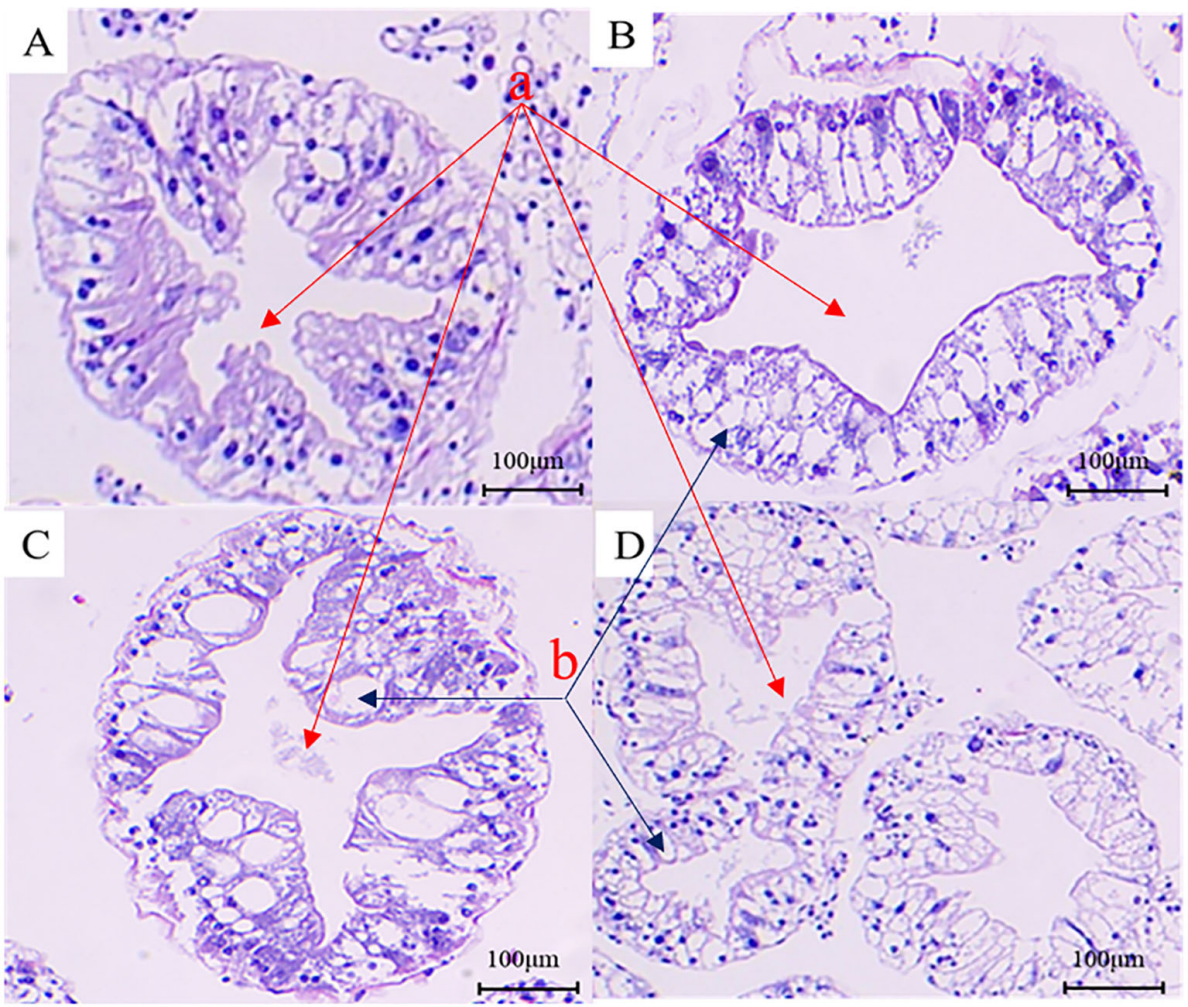
Analysis of histopathological changes in the hepatopancreas tissue of the *Eriocheir sinensis* when challenged with *Vibrio parahaemolyticus* as observed through H&E staining. **(A)** presents the H&E image of the hepatopancreas section from a healthy crab in the control group. **(B–D)** display H&E images of hepatopancreas sections from diseased crabs at 6, 24, and 72 hours post-infection, respectively (a, star-like structure; b, vacuole-like structure).

To analyze the pathogenicity of *V. parahaemolyticus*, we established an animal model to study the infection in Chinese mitten crabs. Subsequently, we assessed the median lethal dose (LD50) within a 24-hour period. The specific results at each time point are presented in **Supplementary Table S1**. The LD50 of this pathogenic strain was calculated to be 1.78×10^6^ CFU/mL using a formula calculation method (Saganuwan, 2016). An animal infection model was established using the LD50 concentration. The changes in the hepatopancreas tissue of clinical animals were analyzed at various time points during the early, middle, and late stages of infection. The control group exhibited good condition and vigor, with crabs displaying active behavior, strong grip in their claw feet, and intact hepatopancreas with clear folds upon dissection. The gills appeared light cyan with neat filaments (**Figures 2A**, **3A**). After 6 hours of infection with *V. parahaemolyticus*, diseased crabs demonstrated significant abnormalities, weakened vitality, and reduced crawling ability. Dissection revealed a dark yellow appearance of the hepatopancreas, cloudy and blackened gills, and dilated stellate structures with contracted folds in the hepatopancreatic tissue (**Figures 2B**, **3B**). At 24 hours post-infection, crabs exhibited low vitality, moderate crawling ability, and a tendency to escape when disturbed. The hepatopancreas appeared significantly orange-yellow with slight erosion, and the gills turned black. Hepatopancreatic tissue displayed enlarged vacuoles, ruptured stellate structures, and continued expansion (**Figures 2C**, **3C**). By 72 hours after infection, the survival rate of diseased crabs was very low. They showed minimal activity, with no tendency to break free when handled. Dissection revealed necrosis in the hepatopancreas, eroded liver tissue with whitish coloration, and completely dark brown gills. The hepatopancreatic ductal structure exhibited increased folding contraction and extensive loss of nuclei (**Figures 2D**, **3D**).

### 3.3 Tissue enzyme viability test

**Figure 4:**
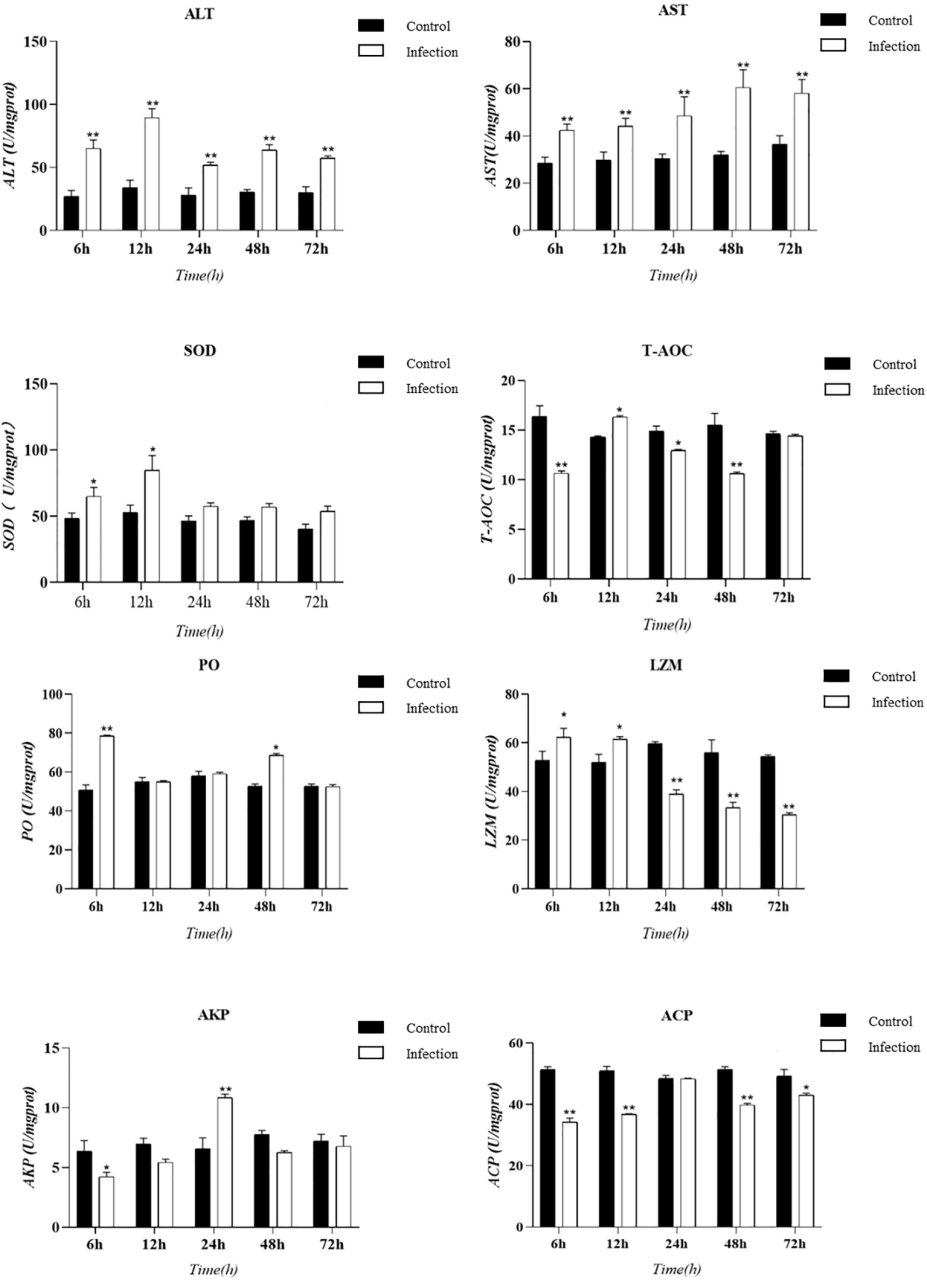
Presents a histogram illustrating the enzyme activity in the hepatopancreas tissue of *Eriocheir sinensis* at various time points following a challenge with *Vibrio parahaemolyticus*. The bar graph displays the mean ± standard error (SE) based on six experimental animals (n = 6). Asterisks denote significant differences between the samples (*P < 0.05; **P < 0.01).

Tissue enzyme activity serves as a critical indicator of the innate immunity in experimental animals. In this study, we utilized an animal infection model to collect hepatopancreas tissue from *E. sinensis* and measured various enzyme activity indicators, including ALT, AST, ACP, LZM, SOD, T-AOC, AKP, and PO. The results are presented as follows: changes in enzyme activities at various time points during infection were observed through the hepatopancreatic immune factor assay (**Figure 4**). Compared to the control group, the levels of ACP and T-AOC were significantly lower (*P* < 0.05). However, there was a significant increase in the activity of SOD and lysozyme (LZM) within the initial 12 hours following infection (*P* < 0.05). Subsequently, LZM activity decreased and was significantly lower than that of the control group (*P* < 0.05). The results from the body fluid enzyme activity factor assay demonstrated significant activation of specific oxidative enzymes and lysozyme during the early phases of infection. Conversely, ALT and AST consistently exhibited significant activation throughout the entire infection cycle (*P* < 0.05).

### 3.4 Gene prediction and annotation

During the process of *E. sinensis* resisting infection by Vibrio parahaemolyticus, the organism’s genes undergo differential transcription. Utilizing an animal infection model, we collected the hepatopancreas from experimental animals exhibiting various clinical symptoms during the infection. We analyzed the differential changes in its transcriptome through RNA-seq sequencing technology. The resulting data is presented as follows: A total of 205,181,269 raw data reads were obtained from the nine samples (**Table 3**). Specifically, 69,600,138 raw reads were from the control group (Es_CG), 68,521,173 from the susceptible group (Es_SC), and 67,059,958 from the disease-resistant group (Es_AI). After screening, 20,215,931 high-quality Clean Reads were obtained, accounting for 98.53% of the total data. For transcript splicing, 68,583,106, 67,510,983, and 66,060,842 Clean Reads were obtained from the control, susceptible, and resistant groups, respectively. The raw data exhibited Q20 > 96.51%, Q30 > 91.56%, and GC content > 50.13%. Using *de novo* assembly with Trinity, a total of 232,924 genes were predicted. The shortest unigene was 301 bp, the longest 30,836 bp, with an average length of 991 bp. The N50 and N90 values were 1464 and 412, respectively (**Table 4**). All sequencing raw data from this study have been deposited in the National Center for Biotechnology Information database under the accession number NCBI: PRJNA1130557.

**Table 3:** Summary of sample sequencing data quality.

Sample	Raw_reads	Clean_reads	Error_rate	Q20	Q30	GC%
Es_CG1	23,307,438	22,942,856	0.03	96.93	92.38	51.39
Es_CG2	23,234,460	22,957,683	0.03	97.13	92.82	51.38
Es_CG3	23,058,240	22,682,567	0.03	97	92.53	51.55
Es_SC1	23,825,811	23,483,810	0.03	96.77	92.09	50.5
Es_SC2	22,328,264	21,996,616	0.03	96.51	91.65	50.75
Es_SC3	22367098	22030557	0.03	96.83	92.29	50.51
Es_AI1	23253911	22910103	0.03	96.97	92.48	50.17
Es_AI2	21736257	21395973	0.03	96.73	92.05	50.13
Es_AI3	22069790	21754766	0.03	96.72	92.02	50.2

Q20: Percentage of overall bases with Phred values greater than 20, where Phred = -10log10(e)

Q30: Percentage of overall bases with Phred values greater than 30, where Phred = -10log10(e)

**Table 4:** Splice length distribution list.

Type	Min length	Mean length	Median length	Max length	N50	N90	Total nucleotides
Transcript	301	1,172	663	30,836	1,884	469	181,070,411
Unigene	301	991	563	30,836	1,464	412	77,692,224

### 3.5 Statistical analysis of differentially expressed genes

**Figure 5:**
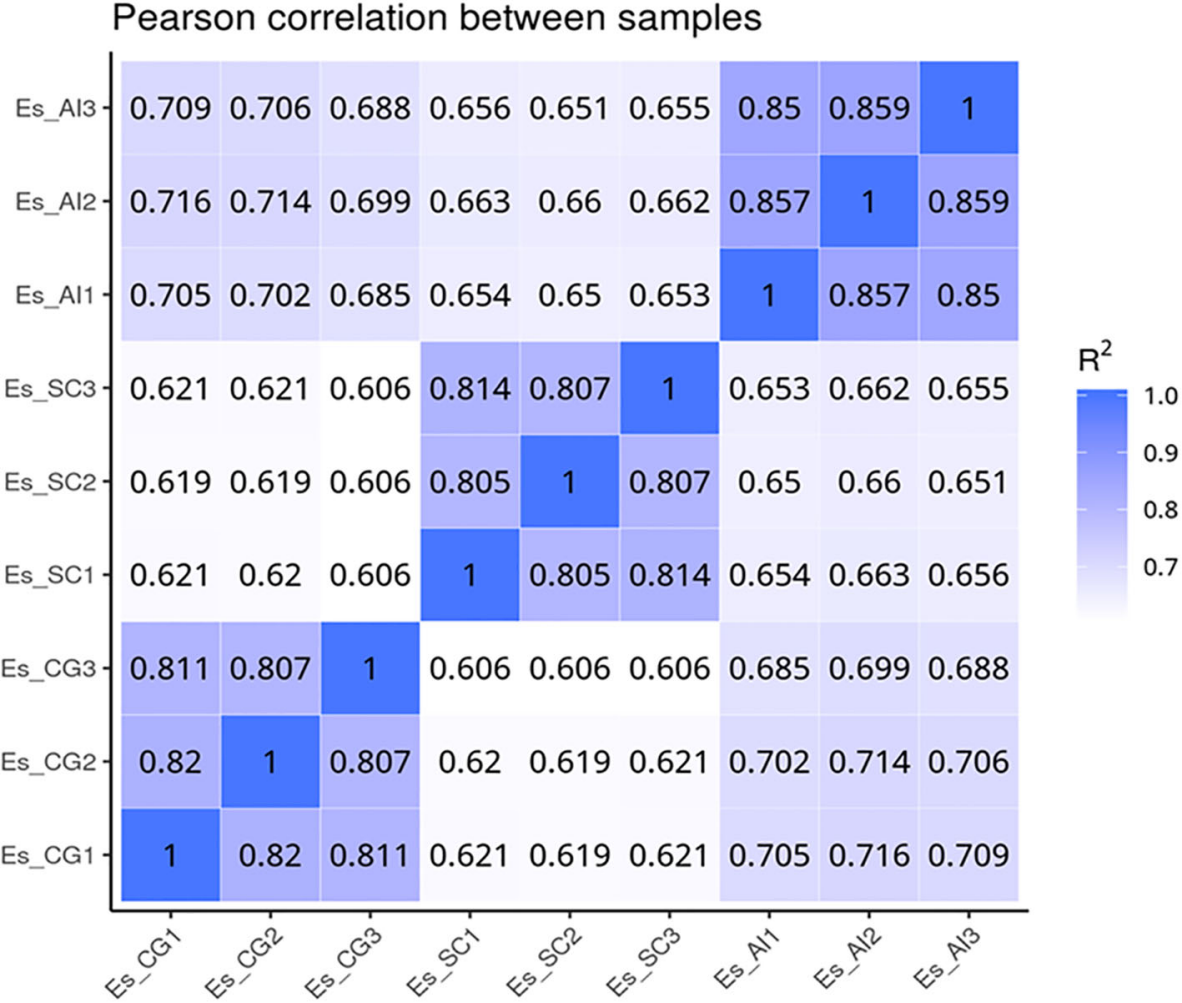
Heat map of correlation coefficients between samples. The x-axis represents the log10 (FPKM+1) of sample 1, while the y-axis represents the log10 (FPKM+1) of sample 2. R^2^ denotes the square of the Pearson correlation coefficient. The correlation of gene expression levels between samples serves as a crucial indicator for assessing the reliability of the experiment and the appropriateness of sample selection. A correlation coefficient closer to 1 signifies a higher similarity in expression patterns between samples. To evaluate this, calculate the intra-group and inter-group correlation coefficients based on the FPKM values of all genes within the samples. Subsequently, represent these correlations in a heat map, which visually illustrates the differences between groups and the replication of samples within each group.

**Figure 6:**
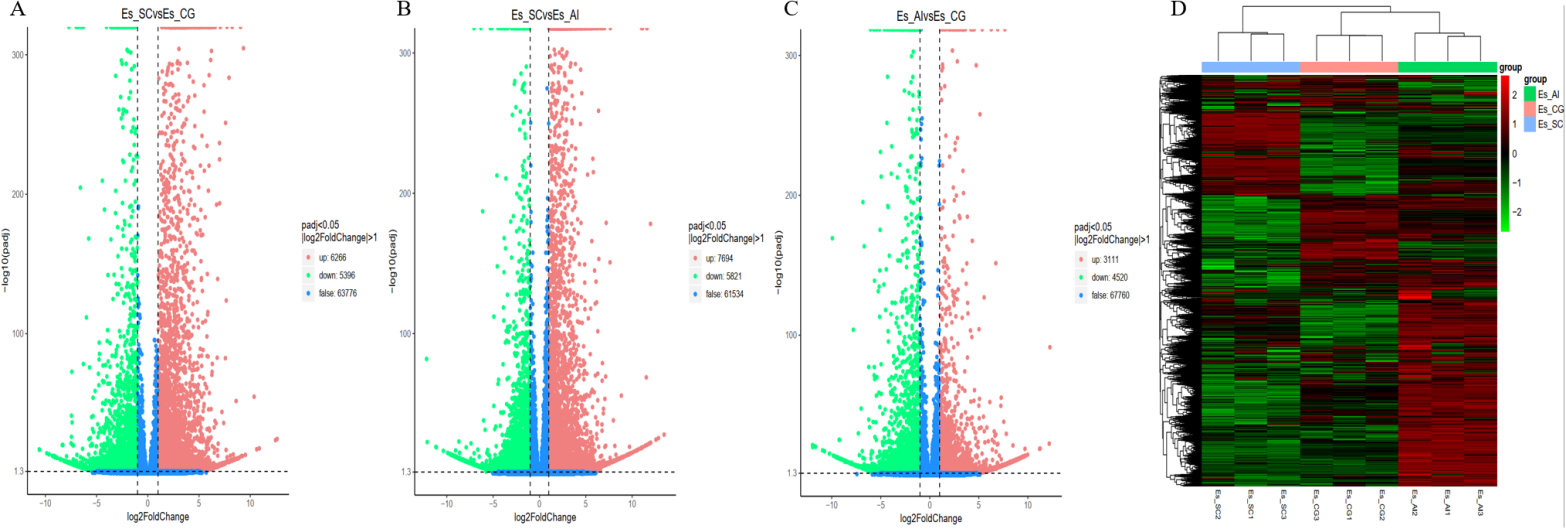
A volcano plot and heat map illustrating the differential gene enrichment of hepatopancreas tissue genes from experimental animals across various groups. **(A)** Comparison of differential gene volcano maps between Es_SC and ES_CG; **(B)** Comparison of differential gene volcano maps between Es_SC and ES_AL; **(C)** Comparison of differential gene volcano maps between Es_AL and ES_CG; **(D)** Heatmap displaying differential gene enrichment for all samples. The volcano plot visually represents the overall distribution of genes exhibiting significant expression differences. The abscissa denotes the fold change in gene expression across different samples (log2FoldChange), while the ordinate indicates the significance level of these expression differences (-log10padj). Up-regulated genes are depicted as red dots, whereas down-regulated genes are represented by green dots. Cluster analysis was conducted on differential gene sets to group genes exhibiting similar expression patterns. We employed hierarchical clustering, a widely used method, to analyze the FPKM values of the genes, normalizing the rows using Z-scores. In the resulting heat map, genes or samples with analogous expression patterns are clustered together. It is important to note that the color in each square does not represent the actual gene expression value; rather, it reflects the normalized values of the expression data, which typically range between -2 and 2.

We utilized data analysis software based on the R programming language to conduct a statistical analysis of differentially transcribed genes across the three groups. For the differential expression of genes in *E. sinensis* infected with *V. parahaemolyticus*, we performed a statistical analysis. Initially, we calculated Pearson’s correlation coefficients using the FPKM values of all genes across the samples. The results, depicted in **Figure 5**, demonstrated strong correlations between the samples (R^2 close to 0.9), indicating the data’s suitability for further analysis. Comparing ES_SC and ESCG, we identified 11,662 genes with differential expression, out of which 6,266 genes were up-regulated and 5,396 genes were down-regulated (**Figure 6A**). Comparing ES_SC and ES_AI, A total of 13,515 genes exhibited differential expression, with 7,694 genes up-regulated and 5,821 genes down-regulated (**Figure 6B**). Likewise, comparing ES_AI and ES-CG, we found 7,631 genes with differential expression, including 3,111 genes that were up-regulated and 4,520 genes that were down-regulated (**Figure 6C**). To visualize the expression patterns of the differentially expressed genes, we generated a heatmap (**Figure 6D**). This heatmap clusters genes with similar expression patterns together. Each square’s color indicates the expression level of the corresponding gene: redder colors denote higher expression levels, while greener colors denote lower expression levels. These analyses provide insights into the transcriptional responses of *E. sinensis* to *V. parahaemolyticus* infection, highlighting significant gene expression changes associated with susceptibility and resistance.

### 3.6 Enrichment of differentially expressed genes

The differential transcription of genes significantly influences the life and metabolic processes of experimental animals. In this study, we enrich and analyze the differentially expressed genes sequenced by RNA-seq using Gene Ontology (GO) enrichment and the KEGG database to elucidate how various hosts resist the invasion of pathogenic microorganisms through their own biological processes. The GO enrichment analysis revealed significant findings in the comparison between ES_AI and ES-CG groups. In terms of biological processes, genes demonstrated enrichment primarily in metabolic processes (149 genes, with 63 up-regulated and 86 down-regulated) and transmembrane transport (305 genes, comprising 152 up-regulated and 153 down-regulated). Cellular component analysis highlighted enrichment in the extracellular matrix (19 genes, with 13 up-regulated and 6 down-regulated). Molecular function analysis indicated significant enrichment in oxidoreductase activity (257 genes, 122 up-regulated and 135 down-regulated). In KEGG pathway analysis, up-regulated genes were notably enriched in pathways such as ribosome (54 genes) and protein processing in the endoplasmic reticulum (38 genes), while down-regulated genes were primarily associated with starch and sucrose metabolism (27 and 24 genes, respectively). These results provide valuable insights into the functional roles of differentially expressed genes in *E. sinensis* during infection with *V. parahaemolyticus*, highlighting key processes and pathways affected by the bacterial infection.

**Figure 7:**
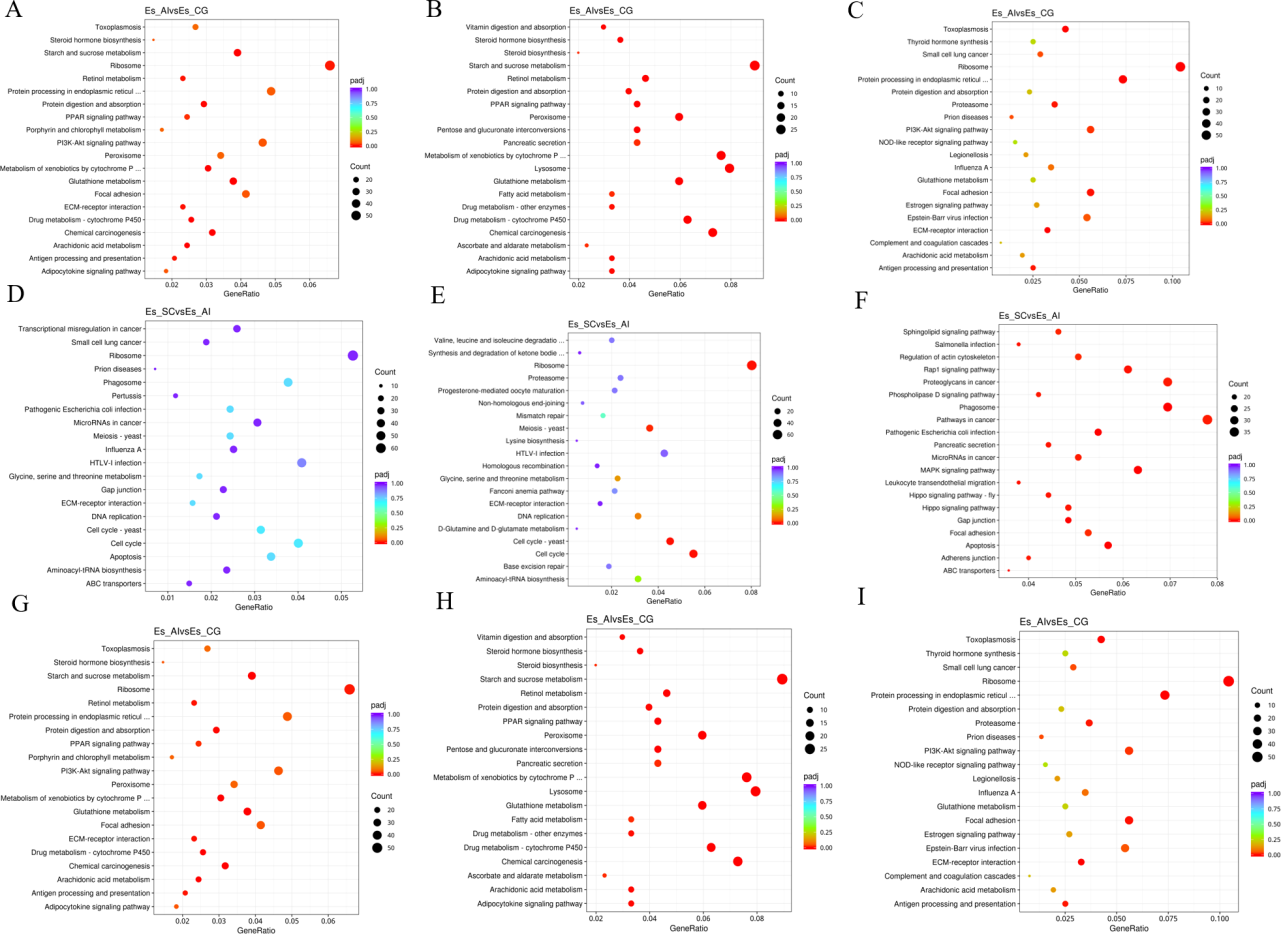
Scatterplot of KEGG pathway enrichment for different comparison combinations. Panels **(A, D, G)** present scatter plots illustrating all differential genes in the comparison of the two groups. Panels **(B, E, H)** display scatter plots for significantly down-regulated genes within the same comparison. Meanwhile, Panels **(C, F, I)** depict scatter plots for significantly up-regulated gene signaling pathways in the two groups. In the KEGG pathway enrichment scatter plot, the vertical axis lists the names of the pathways, while the horizontal axis represents the Rich factor associated with each pathway. The color of each point reflects the magnitude of the q-value, with smaller q-values indicated by colors closer to red. Furthermore, the size of the dots corresponds to the number of genes associated with each pathway.

The comparison between ES_SC and ES_AI demonstrated that the differential genes expressed in biological processes primarily exhibited enrichment in the metabolic process of cellular nitrogen compounds and the biosynthetic process as shown in **Figures 7D–F**. ES_SC had 972 down-regulated genes, while ES_AI had 794 down-regulated genes. Conversely, ES_SC showed 581 up-regulated genes compared to ES_AI’s 689 up-regulated genes. Furthermore, an enrichment of up-regulated genes was observed in the immune system process (20 genes), while 33 genes were down-regulated. Regarding cellular composition, genes showed significant enrichment in intracellular compartments and organelles. Specifically, ES_SC had 795 up-regulated genes compared to 619 in ES_AI. Conversely, ES_SC had 964 down-regulated genes, while ES_AI had 772 down-regulated genes. In terms of molecular functions, genes exhibited prominent enrichment in DNA binding and ion binding. The up-regulated genes were 311 in ES_SC and 433 in ES_AI, while the down-regulated genes were 456 in ES_SC and 433 in ES_AI. Additionally, the KEGG enrichment analysis revealed similar results, indicating that up-regulated genes mainly enriched the phagosome, pathways in cancer, and proteoglycans in cancer pathways, with 33, 37, and 33 enriched genes, respectively. On the other hand, down-regulated genes primarily enriched the cell cycle pathway and the ribosome pathway, with 44 and 64 enriched genes, respectively.

When comparing ES_SC and ES-CG, the differential genes involved in biological processes are primarily enriched in the metabolic process of cellular nitrogen compounds and biosynthesis. There are 732 up-regulated genes and 611 down-regulated genes, along with 662 up-regulated genes and 556 down-regulated genes, respectively. Additionally, there are 22 up-regulated genes and 25 down-regulated genes enriched in the immune system process. In terms of cellular composition, genes are predominantly enriched in intracellular locations and organelles. Specifically, there are 838 up-regulated genes and 655 down-regulated genes enriched, while 648 up-regulated genes and 514 down-regulated genes are observed. Moving on to molecular functions, genes exhibit significant enrichment in DNA binding and ion binding. This enrichment involves 321 up-regulated genes and 507 down-regulated genes for DNA binding, and 302 up-regulated genes and 507 down-regulated genes for ion binding (as shown in **Figures 7G–I**). Regarding the KEGG enrichment analysis, up-regulated genes are primarily enriched in pathways related to cancer, including pathways in cancer itself and Proteoglycans in cancer. The number of enriched genes is 46 and 49, respectively. On the other hand, down-regulated genes show enrichment in pathways related to starch and sucrose metabolism, as well as the lysosome pathway, with 31 and 40 enriched genes, respectively.

**Figure 8:**
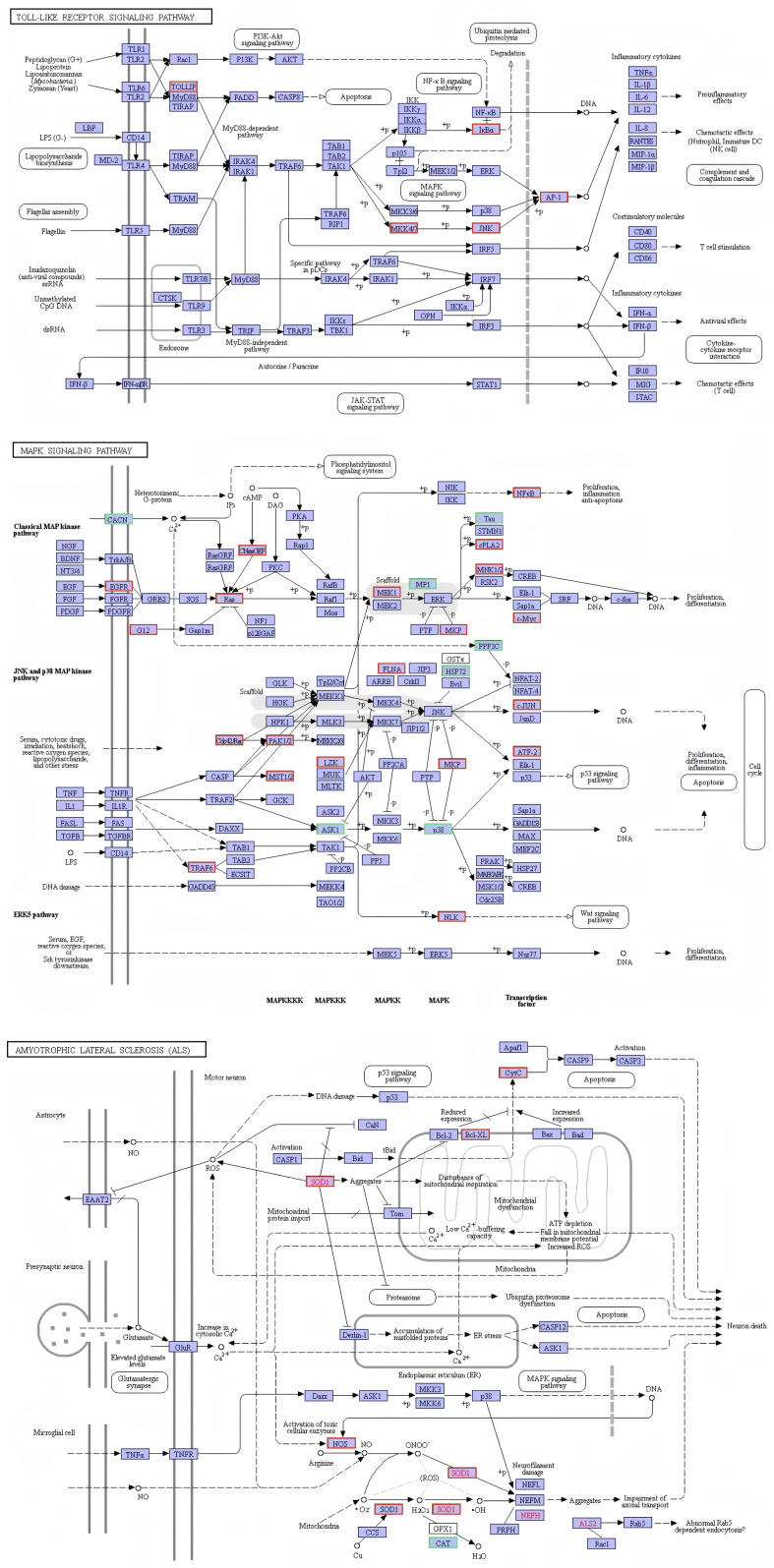
Network diagram of differential gene enrichment signaling pathways. The KEGG database was employed to analyze the localization of signaling pathways enriched in differentially expressed genes and to investigate potential regulatory mechanisms. The red color highlights significantly differentially expressed genes and their respective locations.

The present investigation involved the screening of 691 genes from the immune-related functional classification provided by the KEGG database (**Supplementary Figure S1**). In relation to immunity, a total of 342 differentially expressed genes were discovered. These selected genes exhibited enrichment in diverse KEGG pathways, with a particular emphasis on 22 signaling pathways associated with immune response (**Supplementary Figure S1**). Amongst these pathways, notable considerations included platelet activation (37 genes), leukocyte transendothelial migration (29 genes), Fc gamma R-mediated phagocytosis (26 genes), chemokine signaling pathway (31 genes), toll-like receptor signaling pathway (20 genes), antigen processing and presentation (18 genes), T cell receptor signaling pathway (19 genes), NOD-like receptor signaling pathway (16 genes), B cell receptor signaling pathway (14 genes), Rap1 signaling pathway (38 genes), Fc epsilon RI signaling pathway (14 genes), TNF signaling pathway (16 genes), natural killer cell mediated cytotoxicity (12 genes), RIG-I-like receptor signaling pathway (11 genes), ErbB signaling pathway (15 genes), NF-kappa B signaling pathway (11 genes), Ras signaling pathway (28 genes), inflammatory mediator regulation of TRP channels (14 genes), GnRH signaling pathway (16 genes), cAMP signaling pathway (28 genes), PI3K-Akt signaling pathway (31 genes), and MAPK signaling pathway (27 genes). Several immune-related signaling pathways are graphically displayed in this study. The figure illustrates that infection with *V. parahaemolyticus* triggers the activation of specific genes within the KEGG signaling pathway in the samples analyzed. It is hypothesized that the activation of these genes plays a crucial role in initiating the respective KEGG signaling pathway (**Figure 8**).

### 3.7 Transcriptome differential gene expression validation

**Figure 9:**
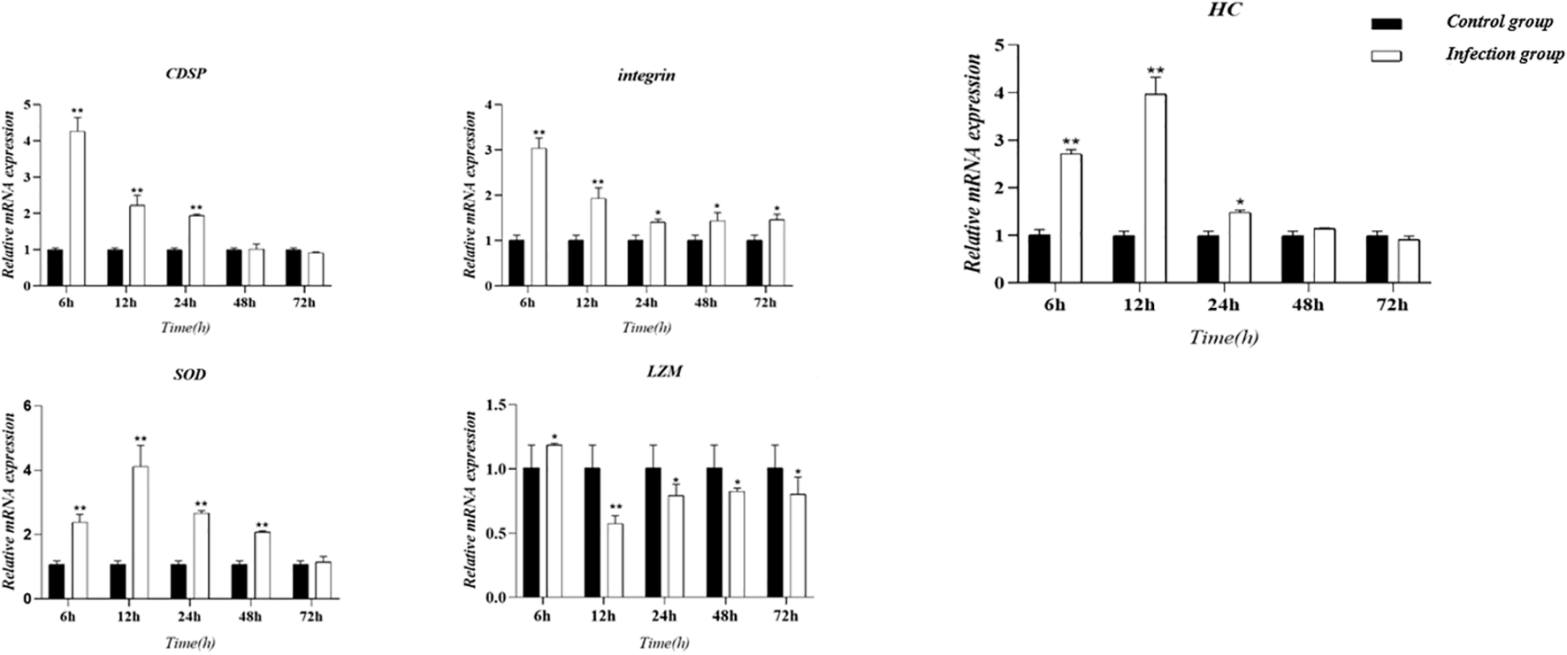
Graph illustrating gene expression trends derived from validated RNA sequencing (RNA-seq) analyses utilizing fluorescence quantification methods. The bar graph displays the mean ± standard error (SE) based on six experimental animals (n = 6). Asterisks denote significant differences between the samples (*P < 0.05; **P < 0.01).

The validation results depicted in **Supplementary Figure S2** demonstrate concordance between RNA sequences and qRT-PCR findings, supporting the accuracy and precision of RNA-Seq in this investigation. Temporal changes in the expression levels of 5 genes were analyzed at different time points (6, 12, 24, 48, and 72 hours) post-infection using qRT-PCR (**Figure 9**). The results revealed a significant increase in the expression of lysozyme-related genes at 6 hours after acute *V. parahaemolyticus* infection. Moreover, the redox-related SOD showed a consistent augmentation throughout the course of the infection, with the highest level of transcription attained at the 12-hour time point. Furthermore, the infection by *V. parahaemolyticus* led to an up-regulation of the integrin gene in the hepatopancreas tissue. The expression level of the integrin gene experienced a considerably significant rise at 6 hours post-infection (*P* < 0.01), followed by a subsequent decline. However, at the 12-hour mark following infection, the integrin gene’s expression remained significantly up-regulated (*P* < 0.01). Between the 24 and 72-hour periods after infection, there was a significant up-regulation of the integrin gene in crab hepatopancreas tissue (*P* < 0.05), persisting at a sustained level below the normal range. Concurrently, the expression level of the hemocyanin HC gene in the hepatopancreas exhibited a notable rise (*P* < 0.01), followed by a subsequent increase. The expression of HC peaked at 12 hours post-infection (*P* < 0.01), after which it began to decline. Between 48 and 72 hours after infection, the HC gene expression returned to levels close to the baseline.

## 4 Discussion

*V. parahaemolyticus* is a highly pathogenic bacterium known for causing significant mortality and economic losses in cultured *E. sinensis* (Liu et al., 2020; Powers et al., 2021). This study focused on *V. parahaemolyticus*-infected *E. sinensis* and involved constructing a transcriptome sequencing library from immune-related tissues in the hepatopancreas. This approach enabled the acquisition of gene sequences and functional information, substantially enriching the transcriptional expression and genetic information database of the crab. The expanded database will facilitate more comprehensive and systematic analyses of various aspects of crab biology, thereby aiding in management and disease control efforts. Additionally, we confirmed that the strains used in our study carry virulence genes such as *Gyr*, *Tna*, *Pyr*, *Rec*, *Lux*, and *priA/B*. Furthermore, these strains showed sensitivity to antibiotics including propoxur, norfloxacin, tetracycline, and piperacillin. Infection of laboratory animals with *V. parahaemolyticus* induced disturbances in genetic material and energy metabolism in the hepatopancreas of the Chinese mitten crab.

The results of our study indicate that this particular strain of *V. parahaemolyticus* causes acute hepatopancreatic necrosis symptoms in *E. sinensis*. These symptoms resemble those observed in Chinese mitten crabs suffering from acute hepatopancreatic necrosis syndrome (AHPNS), a chronic disease affecting the hepatopancreas and energy consumption (Huang et al., 2021). Based on this similarity, we hypothesized that *V. parahaemolyticus* infection in Chinese mitten crabs could lead to increased depletion of hepatopancreatic energy, ultimately resulting in hepatopancreatic failure and necrosis as a pathological symptom. With prolonged infection duration, we observed significant changes in the color of the hepatopancreas in affected crabs, progressing from dark yellow to white. Additionally, the tissue structure exhibited severe necrosis. Pathological tests revealed deformations in the stellate lumen structure of hepatic tubules in the hepatopancreas of infected crabs, alongside the appearance of vacuoles in hepatocytes, followed by gradual lysis. The hepatopancreas serves as a multifunctional organ integrating metabolic and immune functions, making it the primary target tissue for *V. parahaemolyticus* AHPND infection in crustaceans (Zhang et al., 2023; Miao et al., 2024). *V. parahaemolyticus* infection directly caused acute hepatopancreatic injury, suggesting it could contribute to acute hepatopancreatic necrosis in Chinese mitten crabs.

As typical hydrolases in the innate immune system of crustaceans, alkaline phosphatase (ACP), acid phosphatase (AKP), and lysozyme (LZM) primarily function to directly eliminate extracellular invaders. Therefore, they are considered sensitive biomarkers in the context of environmental stress and microbial infections (Hong et al., 2020). Our study observed an increase in the activity of these enzymes during the early stage of infection, followed by inhibition in the late stage. Based on the analysis of enzyme activity measurement results and pathological sections, we speculate that damage to the hepatopancreas tissue may be one of the reasons for the contribute to the observed decrease in enzyme activity. Further experimentation involving pollutant exposure demonstrated a correlation between decreased enzyme activity in fish and reduced activity of liver and blood cells (Liu et al., 2009), indirectly supporting our initial speculation. Alanine aminotransferase (ALT) and aspartate aminotransferase (AST) are both aminotransferases that belong to the same class and are involved in protein metabolism within the body. These enzymes are primarily located in cellular mitochondria. Changes in their activity can indicate alterations in metabolism or tissue damage (Cheng et al., 2020). In our experiments, we observed significant pathological damage to the hepatopancreas of infected crabs. Additionally, the activities of ALT and AST changed over time during infection, suggesting that the protective mechanism of the crab’s hepatopancreas was impacted by *V. parahaemolyticus* infection. This finding supports the idea that the increased mortality rate observed in Chinese mitten crabs after *V. parahaemolyticus* infection is mainly due to hepatopancreatic damage (Khimmakthong and Sukkarun, 2017).

Oxygen free radicals in aquatic animals exist in a dynamic equilibrium, which can be disrupted by bacterial invasion or other stressors, leading to the generation of large amounts of free radicals. The organism has the ability to activate its antioxidant defense system to eliminate reactive oxygen species and prevent oxidative damage. However, excessive oxidative stress can contribute to disease development. SOD is an enzyme that plays a crucial role in the antioxidant system of organisms and serves as an important component of innate immunity in crustaceans (Guo et al., 2021). *In vitro* bacterial agglutination assays of manganese SOD (MnSOD) have demonstrated its ability to agglutinate a wide range of both Gram-negative and Gram-positive bacteria (Li et al., 2021). During infections caused by *V. parahaemolyticus* or *Staphylococcus aureus*, total SOD activity and mRNA levels of MnSOD were significantly increased, indicating the involvement of MnSOD in antimicrobial immunity through regulation of the cellular redox state (Li et al., 2021). It is hypothesized that bacterial infection may induce alterations in the levels of oxygen free radicals in crabs during the initial phases of infection. SOD and glutathione, as primary cellular antioxidants, work to eliminate excess superoxide ions in tissues.

During RNA-seq, analysis of differentially expressed genes using enrichment techniques revealed a significant overrepresentation of immune genes in multiple signaling pathways. These pathways include the Toll-like receptor (TLR), mitogen-activated protein kinase (MAPK), immunodeficiency (IMD), and Janus kinase (JAK)/signal transducer and activator of transcription (STAT) signaling pathways. Innate immunity, a shared characteristic between invertebrates and mammals, is recognized as the primary defense against microbial pathogens (Hoffmann, 2003). In invertebrates, activation of the Toll and IMD pathways occurs upon invasion by pathogenic molecules, triggering a host defense response. Crustaceans also possess crucial innate immune pathways that play pivotal roles in combating microbial infections (Li et al., 2018). When external pathogenic microorganisms breach the physical barriers of crabs, immune signaling pathways such as Toll, IMD, and JAK/STAT are activated. Regulatory effects of innate immunity, including activation of complement and coagulation cascades, phagocytosis, pro-inflammatory signaling cascades, and apoptosis, are essential components of the host immune response. These processes involve multiple elements such as antimicrobial peptides (AMPs), complement-like proteins, and blood cells (Lemaitre, 2004). In invertebrates, various self-defense mechanisms contribute to the innate immune response. In crustaceans, these mechanisms primarily involve AMP-mediated bacterial killing, generation of reactive oxygen species through the prophenoloxidase-activated system, phagocytosis of microorganisms by hemocytes, and the melanin deposition cascade for microorganism entrapment (Tanji and Ip, 2005). Significant differences were observed in the transcription of genes encoding SOD, CDSP, integrin, LZM, HC and their derivatives. Our study provides valuable insights into the activation of signaling pathways during *V. parahaemolyticus* infection in Chinese mitten crabs.

## 5 Conclusion

Following infection with *V. parahaemolyticus*, Chinese mitten crabs primarily exhibit hepatopancreatic necrosis as the predominant clinical manifestation. Transcriptomic analysis of the hepatopancreas from infected crabs revealed successful annotation of a substantial number of genes across seven major databases. A total of 342 genes associated with immune response displayed differential expression, notable enrichment in both the Toll and MAPK pathways. Our findings indicate that activation of the immune defense mechanism in crabs due to by *V. parahaemolyticus* significantly enhances hepatopancreatic function, as well as the activities of antioxidant and immune-related enzymes within the hepatopancreas.

## Data Availability

The original contributions presented in the study are publicly available. This data can be found here: at the National Center for Biotechnology Information (NCBI) under the storage numbers PRJNA902323 and PRJNA1130557.

## References

[B1] Baker-Austin C. Oliver J. D. Alam M. Ali A. Waldor M. K. Qadri F.. (2018). Vibrio spp. infections. Nat. Rev. Dis. Primers 4, 8. doi: 10.1038/s41572-018-0005-8 30002421

[B2] Bolger A. M. Lohse M. Usadel B. (2014). Trimmomatic: a flexible trimmer for Illumina sequence data. Bioinformatics 30, 2114–2120. doi: 10.1093/bioinformatics/btu170 24695404 PMC4103590

[B3] Chen D. Guo L. Yi C. Wang S. Ru Y. Wang H. (2021). Hepatopancreatic transcriptome analysis and humoral immune factor assays in red claw crayfish (Cherax quadricarinatus) provide insight into innate immunomodulation under Vibrio parahaemolyticus infection. Ecotoxicol Environ. Saf. 217, 112266. doi: 10.1016/j.ecoenv.2021.112266 33930770

[B4] Chen D. Wang H. (2022). Redclaw crayfish (Cherax quadricarinatus) responds to Vibrio parahaemolyticus infection by activating toll and immune deficiency signaling pathways and transcription of associated immune response genes. Fish & Shellfish Immunology 127, 611–622. doi: 10.1016/j.fsi.2022.06.069 35809883

[B5] Chen Z. Tayyab M. Yao D. Aweya J. J. Zheng Z. Zhao X.. (2025). Cell death in crustacean immune defense. Rev. Aquaculture 17 (1), e12976. doi: 10.1111/raq.12976

[B6] Cheng C.-H. Ma H.-L. Deng Y.-Q. Feng J. Jie Y.-K. Guo Z.-X. (2020). Effects of Vibrio parahaemolyticus infection on physiological response, histopathology and transcriptome changes in the mud crab (Scylla paramamosain). Fish Shellfish Immunol. 106, 197–204. doi: 10.1016/j.fsi.2020.07.061 32777460

[B7] Coates C. J. Rowley A. F. (2022). Emerging diseases and epizootics in crabs under cultivation. Front. Mar. Sci. 8. doi: 10.3389/fmars.2021.809759

[B8] Davidson N. M. Oshlack A. (2014). Corset: enabling differential gene expression analysis for *de novo* assembled transcriptomes. Genome Biol. 15, 410. doi: 10.1186/s13059-014-0410-6 25063469 PMC4165373

[B9] Davis J. W. Sizemore R. K. (1982). Incidence of Vibrio species associated with blue crabs (Callinectes sapidus) collected from Galveston Bay, Texas. Appl. Environ. Microbiol. 43, 1092–1097. doi: 10.1128/aem.43.5.1092-1097.1982 7103475 PMC244191

[B10] Grabherr M. G. Haas B. J. Yassour M. Levin J. Z. Thompson D. A. Amit I.. (2011). Full-length transcriptome assembly from RNA-Seq data without a reference genome. Nat. Biotechnol. 29, 644–652. doi: 10.1038/nbt.1883 21572440 PMC3571712

[B11] Guo L. Zhou M. Chen D. Yi C. Sun B. Wang S.. (2021). A new insight to characterize immunomodulation based on hepatopancreatic transcriptome and humoral immune factor analysis of the Cherax quadricarinatus infected with Aeromonas veronii. Ecotoxicol Environ. Saf. 219, 112347. doi: 10.1016/j.ecoenv.2021.112347 34044307

[B12] Han J. E. Tang K. F. Tran L. H. Lightner D. V. (2015). Photorhabdus insect-related (Pir) toxin-like genes in a plasmid of Vibrio parahaemolyticus, the causative agent of acute hepatopancreatic necrosis disease (AHPND) of shrimp. Dis. Aquat Organ 113, 33–40. doi: 10.3354/dao02830 25667334 PMC4785170

[B13] Hoffmann J. A. (2003). The immune response of Drosophila. Nature 426, 33. doi: 10.1038/nature02021 14603309

[B14] Hong Y. Huang Y. Huang Z. (2020). Oxidative stress, immunological response, and heat shock proteins induction in the Chinese Mitten Crab, Eriocheir sinensis following avermectin exposure. Environ. Toxicol. 35, 213–222. doi: 10.1002/tox.22858 31617668

[B15] Huang X. Feng Y. Duan H. Zhao L. Yang C. Geng Y.. (2021). Evaluation of pathology and environmental variables contributing to hepatopancreatic necrosis syndrome of Chinese mitten crab, Eriocheir sinensis. Ecotoxicol Environ. Saf. 215, 112157. doi: 10.1016/j.ecoenv.2021.112157 33773151

[B16] Jin Q. Tian G. Wu J. Jiang H. Zhu F. (2020). Identification and characterization of hemocyte microRNAs in mud crab Scylla paramamosain in response to Vibrio parahemolyticus infection. Aquaculture 524, 735288. doi: 10.1016/j.aquaculture.2020.735288

[B17] Khimmakthong U. Sukkarun P. (2017). The spread of Vibrio parahaemolyticus in tissues of the Pacific white shrimp Litopenaeus vannamei analyzed by PCR and histopathology. Microb. Pathog. 113, 107–112. doi: 10.1016/j.micpath.2017.10.028 29056496

[B18] Krantz G. E. Colwell R. R. Lovelace E. (1969). Vibrio parahaemolyticus from the blue crab Callinectes sapidus in Chesapeake Bay. Science 164, 1286–1287. doi: 10.1126/science.164.3885.1286 5770620

[B19] Lee C. T. Chen I. T. Yang Y. T. Ko T. P. Huang Y. T. Huang J. Y.. (2015). The opportunistic marine pathogen Vibrio parahaemolyticus becomes virulent by acquiring a plasmid that expresses a deadly toxin. Proc. Natl. Acad. Sci. U.S.A. 112, 10798–10803. doi: 10.1073/pnas.1503129112 26261348 PMC4553777

[B20] Lee S. Y. Söderhäll K. (2002). Early events in crustacean innate immunity. Fish Shellfish Immunol. 12, 421–437. doi: 10.1006/fsim.2002.0420 12194453

[B21] Lemaitre B. (2004). The road to toll. Nat. Rev. Immunol. 4, 521–527. doi: 10.1038/nri1390 15229471

[B22] Lemaitre B. Hoffmann J. (2007). The host defense of Drosophila melanogaster. Annu. Rev. Immunol. 25, 697–743. doi: 10.1146/annurev.immunol.25.022106.141615 17201680

[B23] Lemaitre B. Kromer-Metzger E. Michaut L. Nicolas E. Meister M. Georgel P.. (1995). A recessive mutation, immune deficiency (imd), defines two distinct control pathways in the Drosophila host defense. Proc. Natl. Acad. Sci. U.S.A. 92, 9465–9469. doi: 10.1073/pnas.92.21.9465 7568155 PMC40822

[B24] Li T. T. Ding Z. F. Pan X. T. Ma F. T. Han K. K. Lei W.. (2018). Characterization of an immune deficiency (IMD) homolog from the oriental river prawn, Macrobrachium nipponense. Fish Shellfish Immunol. 83, S1050464818305412. doi: 10.1016/j.fsi.2018.09.005 30195908

[B25] Li H. Yan Y. Yu X. Miao S. Wang Y. (2011). Occurrence and Effects of the Rhizocephalan Parasite, Polyascus gregarius, in the Chinese Mitten Crab, Eriocheir sinensis, Cultured in a Freshwater Pond, China. J. World Aquaculture Soc. 42, 354–363. doi: 10.1111/j.1749-7345.2011.00474.x

[B26] Li Y. Zhan F. Li F. Lu Z. Shi F. Xu Z.. (2021). Immune function of cytosolic manganese superoxide dismutase from Macrobrachium rosenbergii in response to bacterial infection. Aquaculture 541, 736771. doi: 10.1016/j.aquaculture.2021.736771

[B27] Liu J. Pan L. Q. Zhang L. Miao J. Wang J. (2009). Immune responses, ROS generation and the haemocyte damage of scallop Chlamys farreri exposed to Aroclor 1254. Fish Shellfish Immunol. 26, 422–428. doi: 10.1016/j.fsi.2009.01.002 19141322

[B28] Liu H. Song C. Ning J. Liu Y. Cui Z. (2020). Identification, functional characterization and the potential role of variable lymphocyte receptor EsVLRA from Eriocheir sinensis in response to secondary challenge after Vibrio parahaemolyticus vaccine. Fish Shellfish Immunol. 98, 201–209. doi: 10.1016/j.fsi.2020.01.011 31923564

[B29] Livak K. J. Schmittgen T. D. (2001). Analysis of relative gene expression data using real-time quantitative PCR and the 2(-Delta Delta C(T)) Method. Methods 25, 402–408. doi: 10.1006/meth.2001.1262 11846609

[B30] Loker E. S. Adema C. M. Zhang S. M. Kepler T. B. (2004). Invertebrate immune systems–not homogeneous, not simple, not well understood. Immunol. Rev. 198, 10–24. doi: 10.1111/j.0105-2896.2004.0117.x 15199951 PMC5426807

[B31] Lorgen-Ritchie M. Uren Webster T. McMurtrie J. Bass D. Tyler C. R. Rowley A.. (2023). Microbiomes in the context of developing sustainable intensified aquaculture. 14. doi: 10.3389/fmicb.2023.1200997 PMC10327644 37426003

[B32] Martinez-Urtaza J. Baker-Austin C. (2020). Vibrio parahaemolyticus. Trends Microbiol. 28, 867–868. doi: 10.1016/j.tim.2020.02.008 32931744

[B33] Miao M. Li S. Yu Y. Liu Y. Li F. (2024). Comparative transcriptome analysis of hepatopancreas reveals the potential mechanism of shrimp resistant to Vibrio parahaemolyticus infection. Fish Shellfish Immunol. 144, 109282. doi: 10.1016/j.fsi.2023.109282 38081442

[B34] Newman M. C. Feng S. Y. (1982). Susceptibility and resistance of the rock crab, Cancer irroratus, to natural and experimental bacterial infection. J. Invertebr. Pathol. 40 (1), 75–88. doi: 10.1016/0022-2011(82)90039-8

[B35] Okuda J. Ishibashi M. Hayakawa E. Nishino T. Takeda Y. Mukhopadhyay A. K.. (1997). Emergence of a unique O3:K6 clone of Vibrio parahaemolyticus in Calcutta, India, and isolation of strains from the same clonal group from Southeast Asian travelers arriving in Japan. J. Clin. Microbiol. 35, 3150–3155. doi: 10.1128/jcm.35.12.3150-3155.1997 9399511 PMC230139

[B36] Powers Q. M. Aranguren L. F. Fitzsimmons K. M. McLain J. E. Dhar A. K. (2021). Crayfish (Cherax quadricarinatus) susceptibility to acute hepatopancreatic necrosis disease (AHPND). J. Invertebr Pathol. 186, 107554. doi: 10.1016/j.jip.2021.107554 33596436

[B37] Qin Z. Wang S. Wu Y. Sun J. Zhao F. (2024). Seasonal dynamics of intestinal microbiota in juvenile Chinese mitten crab (Eriocheir sinensis) in the Yangtze Estuary. Front. Cell Infect. Microbiol. 14. doi: 10.3389/fcimb.2024.1436547 PMC11254619 39027136

[B38] Saganuwan S. A. (2016). The new algorithm for calculation of median lethal dose (LD(50)) and effective dose fifty (ED(50)) of Micrarus fulvius venom and anti-venom in mice. Int. J. Vet. Sci. Med. 4, 1–4. doi: 10.1016/j.ijvsm.2016.09.001 30255031 PMC6145044

[B39] Schulenburg H. Boehnisch C. Michiels N. K. (2007). How do invertebrates generate a highly specific innate immune response? Mol. Immunol. 44, 3338–3344. doi: 10.1016/j.molimm.2007.02.019 17391764

[B40] Sizemore R. K. Colwell R. R. Tubiash H. S. Lovelace T. E. (1975). Bacterial flora of the hemolymph of the blue crab, Callinectes sapidus: numerical taxonomy. Appl. Microbiol. 29, 393–399. doi: 10.1128/am.29.3.393-399.1975 1090258 PMC186986

[B41] Sritunyalucksana K. Söderhäll K. (2000). The proPO and clotting system in crustaceans. Aquaculture 191, 53–69. doi: 10.1016/S0044-8486(00)00411-7

[B42] Sun R. Yue F. Qiu L. Zhang Y. Wang L. Zhou Z.. (2013) The CpG ODNs enriched diets enhance the immuno-protection efficiency and growth rate of Chinese mitten crab, Eriocheir sinensis. Fish Shellfish Immunol. 35, 154–160. doi: 10.1016/j.fsi.2013.04.015 23623940

[B43] Tanji T. Ip Y. T. (2005). Regulators of the Toll and Imd pathways in the Drosophila innate immune response. Trends Immunol. 26, 193–198. doi: 10.1016/j.it.2005.02.006 15797509

[B44] Thitamadee S. Prachumwat A. Srisala J. Jaroenlak P. Salachan P. V. Sritunyalucksana K.. (2016). Review of current disease threats for cultivated penaeid shrimp in Asia. Aquaculture 452, 69–87. doi: 10.1016/j.aquaculture.2015.10.028

[B45] Tian L. Fang G. Li G. Li L. Zhang T. Mao Y. (2024). Metagenomic approach revealed the mobility and co-occurrence of antibiotic resistomes between non-intensive aquaculture environment and human. Microbiome 12, 107. doi: 10.1186/s40168-024-01824-x 38877573 PMC11179227

[B46] Tong R. Pan L. Zhang X. Li Y. (2022). Neuroendocrine-immune regulation mechanism in crustaceans: A review. 14, 378–398. doi: 10.1111/raq.12603

[B47] Tran L. Nunan L. Redman R. M. Mohney L. L. Pantoja C. R. Fitzsimmons K.. (2013). Determination of the infectious nature of the agent of acute hepatopancreatic necrosis syndrome affecting penaeid shrimp. Dis. Aquat Organ 105, 45–55. doi: 10.3354/dao02621 23836769

[B48] Vandeputte M. Coppens S. Bossier P. Vereecke N. Vanrompay D. (2024). Genomic mining of Vibrio parahaemolyticus highlights prevalence of antimicrobial resistance genes and new genetic markers associated with AHPND and tdh + /trh + genotypes. BMC Genomics 25, 178. doi: 10.1186/s12864-024-10093-9 38355437 PMC10868097

[B49] Vazquez L. Alpuche J. Maldonado G. Agundis C. Pereyra-Morales A. Zenteno E. (2009). Review: Immunity mechanisms in crustaceans. Innate Immun. 15, 179–188. doi: 10.1177/1753425909102876 19474211

[B50] Wang W. (2011). Bacterial diseases of crabs: a review. J. Invertebr Pathol. 106, 18–26. doi: 10.1016/j.jip.2010.09.018 21215353

[B51] Wu Y. M. Yang L. Li X. J. Li L. Wang Q. Li W. W. (2017). A class B scavenger receptor from Eriocheir sinensis (EsSR-B1) restricts bacteria proliferation by promoting phagocytosis. Fish Shellfish Immunol. 70, 426–436. doi: 10.1016/j.fsi.2017.09.034 28916359

[B52] Xin Z.-Z. Zhang X.-T. Zhou M. Chen J.-Y. Zhu Z.-Q. Zhang J.-Y. (2024). Differential molecular responses of hemolymph and hepatopancreas of swimming crab, Portunus trituberculatus, infected with Ameson portunus (Microsporidia). Fish Shellfish Immunol. 145, 109324. doi: 10.1016/j.fsi.2023.109324 38134977

[B53] Zhang X. Han Z. Chen F. Sun X. Sun J. (2023) Transcriptomic analysis of the hepatopancreas of white shrimp Penaeus vannamei following experimental infection with the Vibrio parahaemolyticus TJA114. Aquaculture 574, 739662. doi: 10.1016/j.aquaculture.2023.739662

[B54] Zhang Y. Lai Y. Zhou X. Zhu F. (2022). The Role of microRNA-133 in Hemocyte Proliferation and Innate Immunity of Scylla paramamosain. 12. doi: 10.3389/fimmu.2021.812717 PMC8828940 35154084

[B55] Zhang Y. Li Z. Kholodkevich S. Sharov A. Chen C. Feng Y.. (2020). Effects of cadmium on intestinal histology and microbiota in freshwater crayfish (Procambarus clarkii). Chemosphere 242, 125105. doi: 10.1016/j.chemosphere.2019.125105 31675589

[B56] Zhang S. Shi Z. Zhang J. Bonami J. R. (2004). Purification and characterization of a new reovirus from the Chinese mitten crab, Eriocheir sinensis. J. Fish Dis. 27, 687–692. doi: 10.1111/j.1365-2761.2004.00587.x 15575876

