# Supplementary material for: Comparative transcriptomic and molecular biology analyses to explore potential immune responses to Vibrio parahaemolyticus challenge in Eriocheir sinensis
Source: Front Cell Infect Microbiol. 2024 Dec 20;14:1456130. doi: 10.3389/fcimb.2024.1456130 (PMC11695290; doi:10.3389/fcimb.2024.1456130)
Supplement: Supplementary file 1 [file Table1.docx]

**
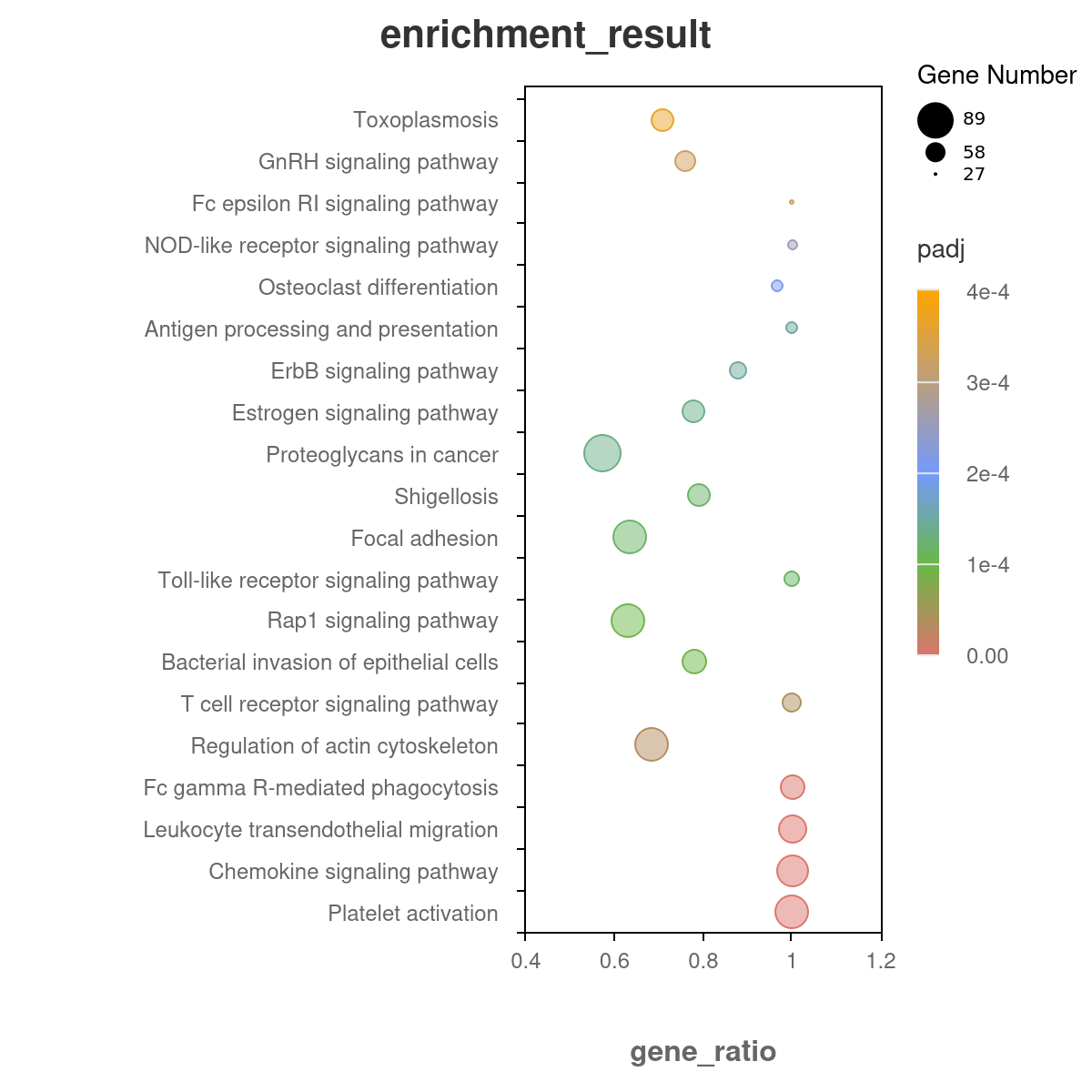
**

**Figure S1 Major signaling pathways for immune gene enrichment**


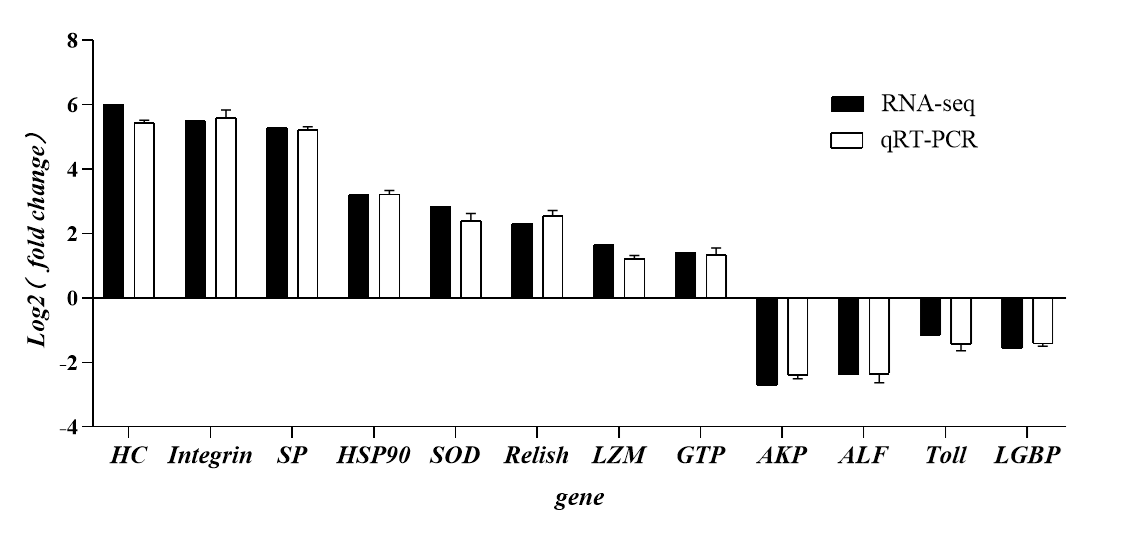


**Figure S2 Transcriptome differential gene expression validation results**

**Table S1 Cumulative mortality in the *V. parahaemolyticus* test for *E. sinensis***

| Time（h） | 6h | 12 h | 24 h | 48 h | 72 h |
| --- | --- | --- | --- | --- | --- |
| Bacterial concentration  （CFU/mL） |  |  |  |  |  |
| PBS | 0 | 0 | 0 | 0 | 0 |
| 10^5^ | 0 | 0 | 5 | 5 | 5 |
| 10^6^ | 0 | 15 | 25 | 40 | 45 |
| 10^7^ | 10 | 60 | 60 | 90 | 90 |
| 10^8^ | 90 | 100 | 100 | 100 | 100 |
